# Predictors of Understaging with EUS and PET-CECT in Early Esophageal Carcinoma

**DOI:** 10.1007/s12029-024-01147-y

**Published:** 2024-12-11

**Authors:** Karthik Venkataramani, Sabita Jiwnani, Devayani Niyogi, Virendrakumar Tiwari, C. S. Pramesh, George Karimundackal

**Affiliations:** 1 Department of Surgical Oncology, Tata Memorial Hospital & Homi Bhabha National Institute, Dr. E. Borges Road, Parel, Mumbai 400012 India; 2 Director of Thoracic Surgery, Max Nanavati Super Speciality Hospital, Mumbai, India

**Keywords:** Esophagectomy, Cancer staging, Endoscopic ultrasonography

## Abstract

**Background:**

The clinicoradiological staging for esophageal cancer is fraught with variable accuracy, potentially depriving patients who have been understaged of the benefit of neoadjuvant therapy, which has been shown to improve long-term survival in locally advanced malignancies. It is imperative to identify these high-risk tumors for tailored treatment.

**Methods:**

Retrospective analysis of a prospective database of patients undergoing esophagectomy for carcinoma esophagus between 2011 and 2019. Patients with clinicoradiological early-stage esophageal carcinoma (T1/2 and N0), staged with EUS and fluoro-deoxy-glucose positron emission tomography with contrast-enhanced computed tomography (FDG PET-CECT), and undergoing upfront surgery were included. Demographic profile, staging, perioperative outcomes, and follow-up data were extracted from electronic records and analyzed using SPSS 26.0.

**Results:**

During this period, we performed 1496 esophagectomies, of which 68 patients (4.5%) underwent upfront surgery for early-stage tumors. The overall concordance between clinical and surgical staging was 55.8%. The positive predictive value (PPV) of EUS for T1, T2, and N0 was 81.6%, 46.7%, and 82.4%, respectively, with 10.2% and 17% upstaging to T3 and N + , respectively. On multivariate analysis, T2 on EUS and tumors longer than 3.5 cm and having standardized uptake value (SUVmax) > 3.05 on FDG PET were strong predictors of stage migration. The 3-year overall survival (OS) of the entire cohort was 74.2%, while those who were understaged had a worse outcome, with a 3-year survival of 48.2%.

**Conclusion:**

Endoscopic T2 stage, length more than 3.5 cm, and SUVmax more than 3.05 are associated with significant understaging and hence should be considered for neoadjuvant therapy.

## Introduction

Carcinoma esophagus is the eighth most common solid tumor and accounts for 5.5% of all cancer-related deaths [1]. The overall survival across stages is a dismal 20% at 5 years; however, survival ranging from 65 to 70% has been demonstrated in patients with early-stage disease subjected to curative resection [2, 3]. The diagnosis of early-stage disease is around 40% [4] in the western population possibly due to increased awareness about the symptomatology and ease of access to endoscopic evaluation for upper gastrointestinal complaints when deemed necessary. In the Indian subcontinent, locally advanced and disseminated stages at presentation account for more than 70% [5, 6]. The stage at presentation is an important factor in predicting long-term survival, and so is the delivery of curative intent treatment. At present, contrast-enhanced computed tomography (CECT) combined with positron emission tomography (PET), esophagogastroduodenoscopy, and endoluminal ultrasound (EUS) are important components of pretreatment staging [7]. But these modalities are fraught with their own disadvantages with varying accuracy especially in early-stage tumors. EUS aids in T and N staging in esophageal cancers but is highly sensitive for T3 and T4 stages and less so for T1 and T2 [8]. EUS also allows for sampling of suspicious regional nodes hence its accuracy for N staging is better than T staging [9]. Studies have shown factors like depth of tumor invasion, tumor size, location, grade, and obstructive lesions to hamper the accuracy of the staging investigations resulting in significant under or over-staging [10–12].

The incorporation of neoadjuvant therapy (neoadjuvant chemotherapy or neoadjuvant chemoradiotherapy) has been shown to significantly improve outcomes by downstaging locally advanced tumors, ensuring a higher number of R0 resections, reducing disease recurrence and prolonging survival [13–16]. This has been shown in both adeno- as well as squamous carcinomas. Given the limitations of the current staging modalities, there is a considerable proportion of patients who are understaged and hence are deprived of neoadjuvant therapy. Current evidence on adjuvant therapy in upfront operated locally advanced carcinoma esophagus is not as robust as that from neoadjuvant trials and is yet not the standard of care [17–19].

The primary outcome of this study was to determine the accuracy of current staging modalities in early-stage carcinoma esophagus. We also aim to find the factors associated with understaging in early esophageal carcinoma and the long-term outcomes of the understaged tumors.

## Materials and Methods

**Fig. 1:**
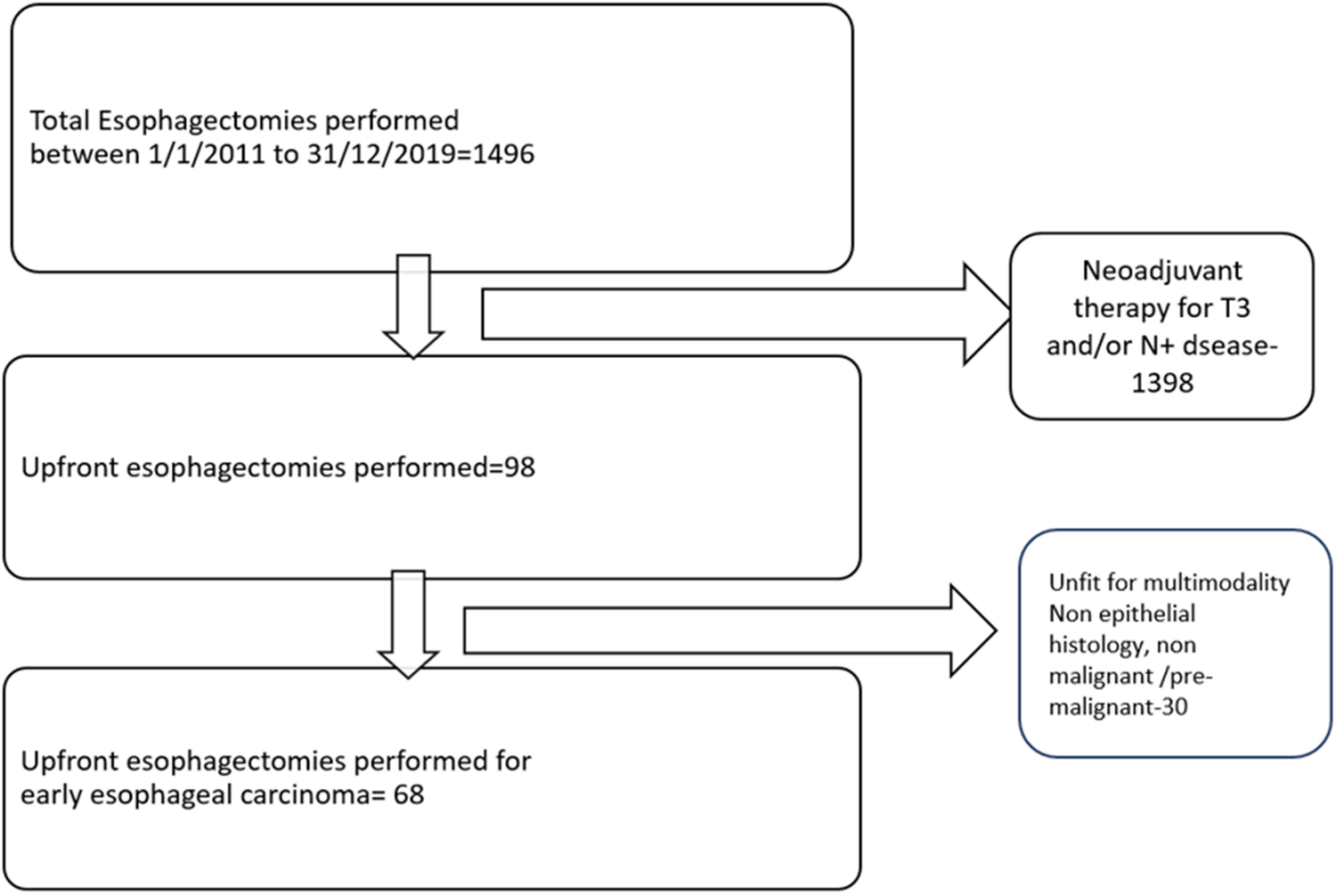
Study schema

We performed a retrospective analysis of a prospectively maintained database of esophageal cancers undergoing curative resection at a tertiary referral cancer center (Fig. 1). Patients with early-stage esophageal carcinoma (T1/T2 and N0) were identified with EUS after ruling out distant metastasis with an 18-fluro deoxy-glucose PET-CECT. They were subjected to esophagectomy after a multidisciplinary tumor board discussion which decided on the extent of surgery and the approach. Patients with non-invasive disease, non-epithelial histology, endoscopic resections, and locally advanced tumors subjected to upfront surgery in view of limited fitness for multimodality therapy were excluded for analysis. The study period was between January 2011 and December 2019.

Data with respect to demographics, pretreatment tumor characteristics, staging, perioperative outcomes, surgical pathology (8th AJCC edition), and follow-up were obtained from the database and electronic medical records and analyzed using SPSS 26.0. The pretreatment staging was compared with the surgical pathology and standard measures of accuracy (sensitivity, specificity, and predictive values) were used for the same. Univariate analysis was carried out to identify the predictors of understaging and a multivariate analysis was done using logistic regression using the stepwise backward method (Wald). Coefficients obtained from the logistic regression analysis were expressed in terms of odds of event occurrence (odd ratio—OR) with 95% confidence intervals (95% CI). *p* < 0.05 was considered statistically significant.

## Results

A total of 1496 esophagectomies were performed between 1 January 2011 and 31 December 2019 of which 68 (4.5%) were upfront surgery for early-stage tumors. Men accounted for 66% (*n* = 45) of the study group with a mean age of 54.2 years (range 34–71 years) (Table 1). Tumors were located in the middle third in 52.9% (*n* = 36) and lower third in the remaining (*n* = 32). Squamous carcinoma accounted for the majority of the cases (*n* = 63, 92.6%) while adenocarcinoma was seen in only 7.4% (*n* = 5). One-fourth (*n* = 17, 25%) of the tumors were poorly differentiated, and the remaining (*n* = 51) were moderately or well differentiated. The pretreatment stage was T2N0 in 44% (*n* = 30) while 56% (*n* = 38) were T1N0. The mean longitudinal tumor size was 2.9 cm.

**Table 1:** Demographic and treatment profile of patients with early-stage tumors

*N*	68
Sex	
Men	45 (66.1%)
Women	23 (33.9%)
Age	Mean 54.2 years
	Range (34–71 years, SD 8.10)
Location	
Upper 1/3	0
Middle third	36 (52.9%)
Lower 1/3 including gastro esophageal junction	32 (47.1%)
Histology	
Squamous	63 (92.6%)
Adenocarcinoma	5 (7.4%)
Grade	
Well differentiated	5 (7.3%)
Moderately differentiated	46 (67.7%)
Poorly differentiated	17 (25%)
Tumor length	Mean 2.9 cm
	Range (1–7 cm)
Surgery	
Trans thoracic	63 (94.1%)
Trans hiatal	4 (4.4%)
Thoracoabdominal	1 (1.5%)
Approach	
Open	15
Laparoscopic	44
Robotic	9
Lymphadenectomy	
2-field	49
3-field	14
Abdominal	5
Mean lymph nodal yield	23 (11–74)
Circumferential resection margin positivity	4 (5.68%)
Lymphovascular invasion (LVI)	
Present	11 (16.2%)
Absent	57 (83.8%)

Transthoracic total esophagectomy was the most common surgery performed (*n* = 64), and the rest underwent esophagectomy via the trans hiatal (*n* = 3) or thoracoabdominal approach (*n* = 1). The majority of the patients, i.e., 54 out of 64 (84.3%), underwent minimally invasive surgery. Major postoperative complications, as per Clavien Dindo classification grading (grade > IIIa), were seen in 24.9% (20/68). The 30-day post-operative mortality was 4.4% (3/68). The median ICU stay was 1 day (IQR 0–2 days) and the median hospital stay was 12 days (IQR 9–15 days). Circumferential resection margin was positive in 5.8% (4/68). The mean lymph nodal yield was 23 (range 11–74). Adjuvant radiotherapy was offered in only four of the upstaged tumors in view of high nodal burden and CRM positivity.

The specificity, sensitivity, positive predictive value, and negative predictive value of EUS for T1, T2, and N staging are mentioned in Tables 2 and 3. EUS fared worse in the staging of T2 tumors with a poor PPV of 46.7% (overstaged 33% and understaged 20%) while fared much better with respect to T1 tumors with a PPV of 81.6% (understaged 18.4%). With respect to N staging, the PPV was 82.4% (understaged 17.4%); given the study design of including only N0 status, estimating specificity was not possible. Overall, 10.2% (*n* = 7) of T1/T2 tumors were upstaged to T3 after surgery while 17.6% (*n* = 12) of N0 were upstaged to N + status. Overall, 20.5% (*n* = 14) of the patients were upstaged to T3 or N + after surgery.

**Table 2:** Tumor- and nodal-stage clinicopathological correlation

T stage			
	pT1	pT2	pT3
cT1 (38)	31	6	1
cT2(30)	10	14	6
N stage			
	pN0		pN +
N0 (68)	56		12

**Table 3:** Diagnostic accuracy for tumor and nodal stage for EUS

Factor	Sensitivity	Specificity	Positive predictive value	Negative predictive value
T1	75.6% (59.7%–87.64)	74.1% (53.7%–88.9%)	81.6% (69.6%–89.6%)	66.7%( 52.74%–78.2%)
T2	70% (45.72%–88.1%)	66.7% (51.6%–79.6%)	46.7% (34.9%–58.9%)	84.2%(72.6%–91.5%)
N0	100%(93.6%–100%)	0	82.4% (82.35%–82.35%)	0

### Factors Predicting Understaging

**Fig. 2:**
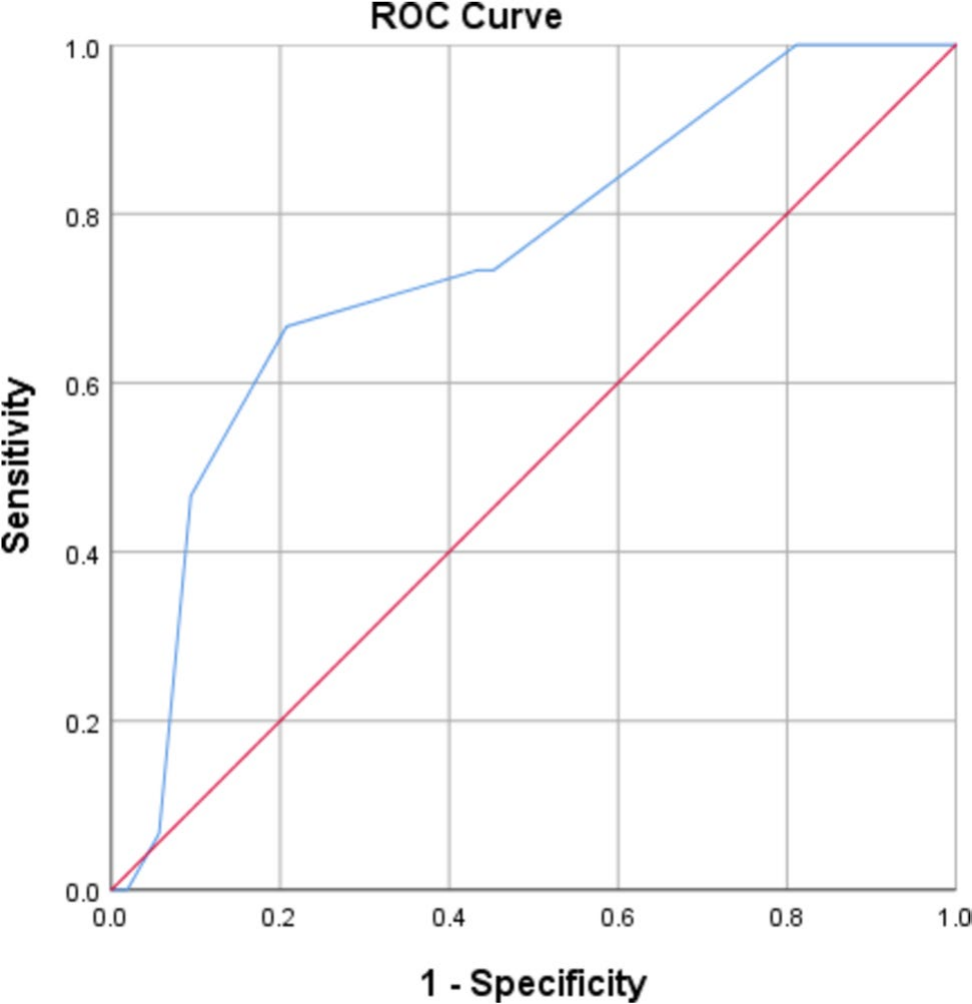
ROC curve showing the association between tumor length and upstaging

**Fig. 3:**
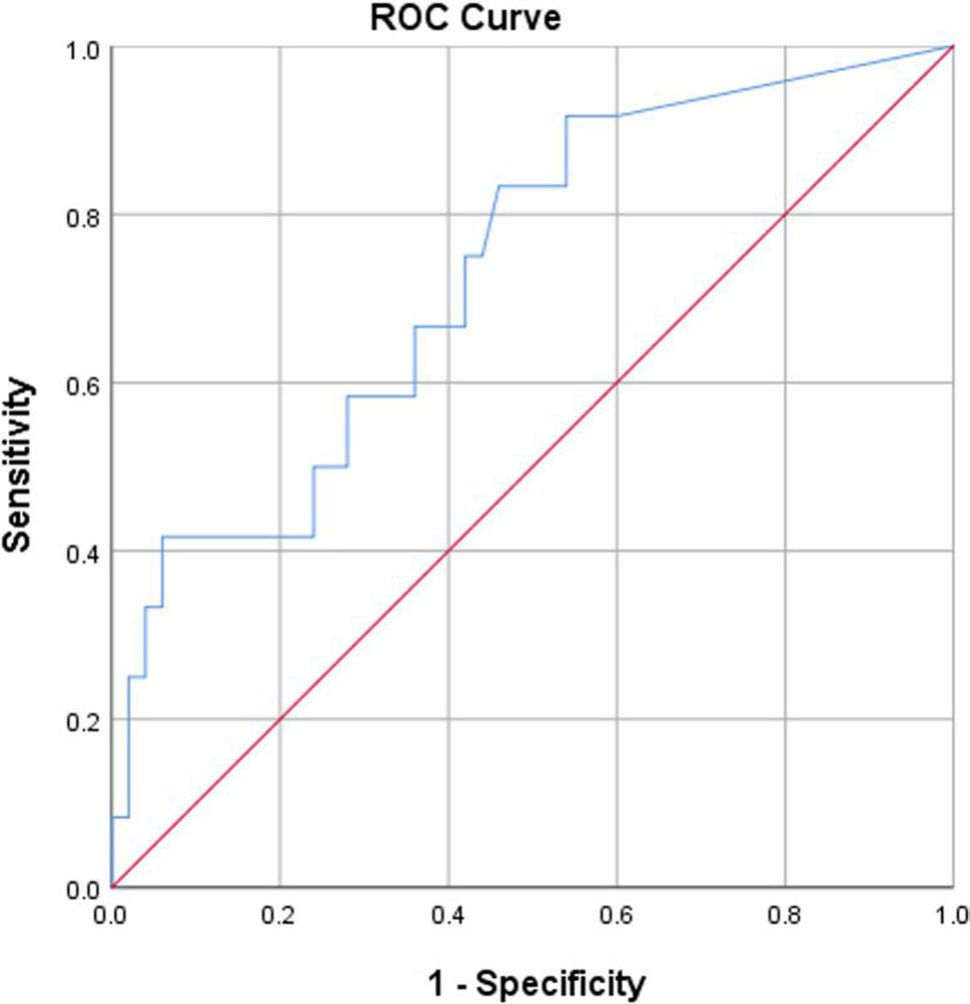
ROC curve showing the association between SUVmax and upstaging

A ROC curve (Figs. 2 and 3) showed a tumor size > 3.5 cm to predict tumor understaging with an AUC of 0.74 (95% CI 0.60–0.889, *p* 0.004) and a SUVmax value of > 3.05 to predict under staging with an AUC of 0.73 (95% CI 0.57–0.88, *p* 0.014).

Multivariate logistic regression was done to identify the factors predicting pre-operative understaging, and tumor size > 3.5 cm, SUVmax > 3.05, and T2 on EUS were significant predictors of understaging (Table 4).

**Table 4:** Multivariate analysis of factors predicting understaging

Factor	χ^2^	Univariate analysis	Multivariate analysis		
			OR	95% CI	*p*-value
Longitudinal tumor size > 3.5 cm	22.3	0.004	2.20	1.15–4.17	0.01
SUVmax > 3.05	55.47	0.015	11.2	1.07–17.8	0.043
T2 on EUS	12.37	0.001	24.8	2.3–261.09	0.007
Poorly differentiated tumor	3.88	0.14	2.4	0.44–13.2	0.25
Squamous histology	0.001	0.15	1.2	0.67–24.5	0.77
Middle third versus lower third	0.720	0.29	0.98	0.16–6.0	0.96
Lymphovascular invasion	1.99	0.15	0.99	0.12–8.02	0.93

### Survival

**Fig. 4:**
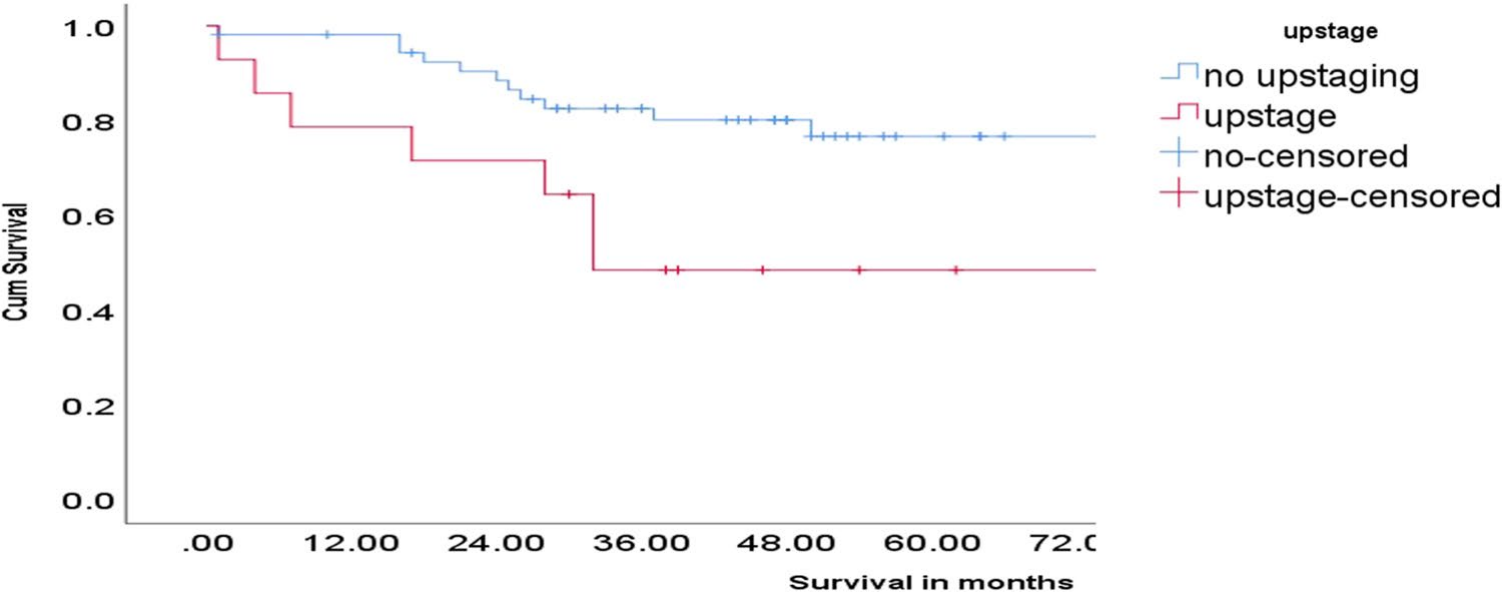
Overall survival outcomes with stage migration on histopathology

**Fig. 5:**
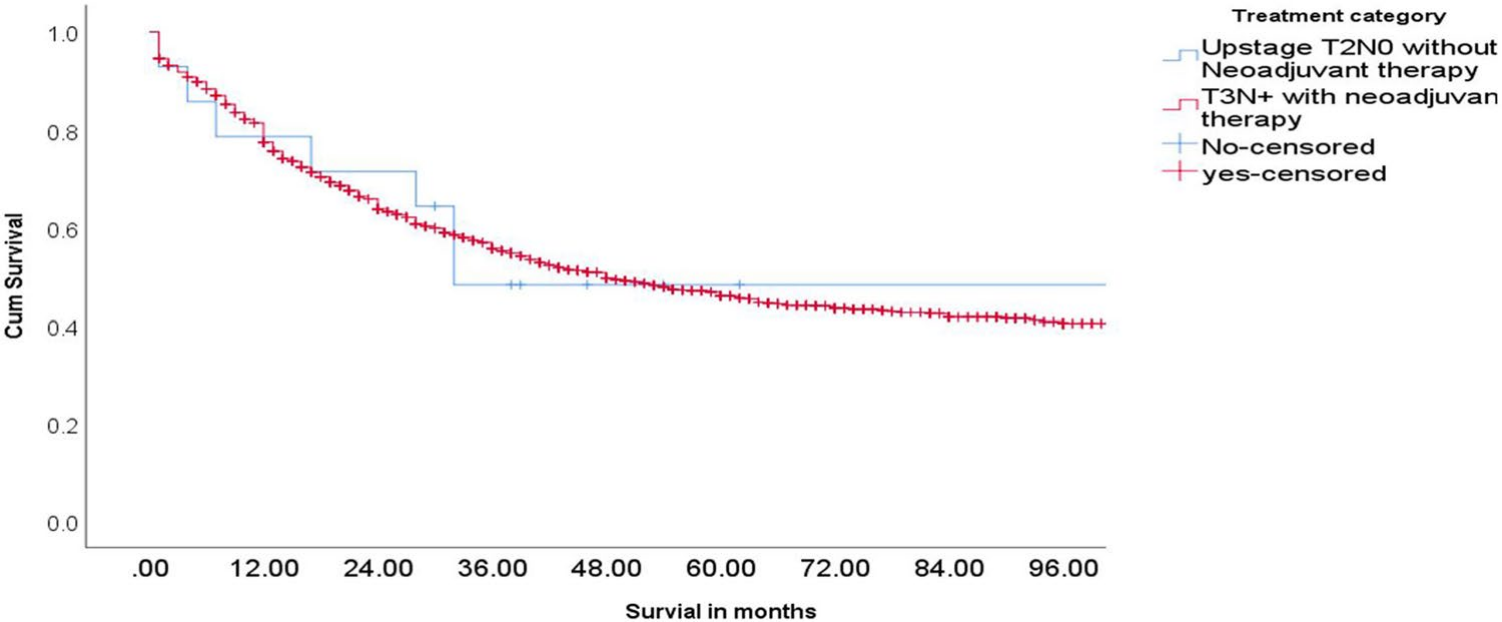
Overall survival outcomes in upstaged T2N0 and upfront locally advanced receiving neoadjuvant therapy

After a median follow-up of 58 months (follow-up cutoff—1 August 2024), the median OS was not reached. At the end of the follow-up period, 26.5% (*n* = 18) of the patients had died due to disease, 4.5% (*n* = 3) were alive with disease, and the rest were disease-free (*n* = 47). The 3-year OS of the population was 74.2%. Subgroup analysis revealed that the patients who were upstaged to T3 or N + status had a 3-year OS of 48.2% (95% CI, 20–71%) while the patients who remained as early-stage tumors after surgery had a 3-year OS of 81.3% (95% CI 68.1–89.4%) as shown in Fig. 4. During the same period, patients with locally advanced disease (≥ T3/N +) treated with neoadjuvant therapy followed by surgery at our institute had a 3-year OS of 56.3% (95% CI 52.9–58.4%) (Fig. 5) despite having a higher pre-treatment radiological tumor burden.

## Discussion

The diagnosis of esophageal carcinoma in the early stage is on the rise; however, it accounts for only a small proportion in our study which is reflective of the existing pattern of the disease and referral in India [3–6, 20]. Squamous cancers continue to be the most common histology in both early- and advanced-stage tumors in the Indian subcontinent in comparison with the western population where adenocarcinoma is the predominant type. Though location or histology has failed to be associated with understaging in early esophageal carcinoma in our study, the gastro-esophageal junctional location was a factor determining understaging in their study by Nucci et al. [21].

The concordance for the clinical stage with the final pathological stage for early-stage esophageal cancer on EUS was 56% which is similar to other reported retrospective studies [11, 22, 23]. The accuracy for diagnosing T2 lesions was 67.6%% (overstaging, 33% and understaging, 20%). Both overstaging and understaging have been reported to be concerns with EUS [8, 23, 24]. Though 33% of T2 tumors were overstaged, surgery remained the treatment of choice in these lesions as they were not amenable to endoscopic resections. Attempting endoscopic resection prior to definitive treatment to know the exact T stage and presence of high-risk features in these T2 tumors has been tried but is not widely accepted [25, 26].

Lymphovascular invasion has been associated with increased risk of nodal metastasis in early esophageal cancers thereby contributing to upstaging; however, it was not a predictor in our study [22, 27].

The PPV for N stage was 82.4%, and of all the upstaged patients (*n* = 14), the majority had nodal upstaging (12/14) where neoadjuvant therapy potentially could have improved outcomes. The routine use of FNA fine needle aspiration cytology for N staging has been shown to improve the accuracy of EUS to above 90% in reported studies [8].

Longitudinal tumor size has been shown in various studies to predict nodal metastasis and long-term outcomes in patients with early-stage disease [10, 28–32], where authors have recommended using tumor size as a part of future AJCC staging [30, 31]. The majority of studies have shown tumor size of greater than 3 cm to be a strong predictor of nodal metastasis in early-stage tumors [10, 22, 33]. NCCN recommends neoadjuvant chemoradiation for cT2N0 tumors with tumor lengths greater than 3 cm [7].

The maximum standardized uptake value in PET-CECT has been shown to predict the T stage with a cutoff value of 4.4 to differentiate the early T stage from the locally advanced [30]. Increasing SUVmax values has also been shown to be a poor predictor for recurrence-free survival [34]. Our study showed an SUVmax value of > 3.05 to be a predictor of understaging with a sensitivity of 88.9%. Tumors with higher SUVmax are shown to have aggressive biology with higher nodal metastasis and poor survival [17, 35]. SUVmax is likely to be a more objective parameter and should be considered one of the factors in deciding neoadjuvant treatment for early-stage esophageal cancers.

Patients with upstaged tumors are shown to have poor survival when compared with patients of similar stages receiving neoadjuvant therapy from large database studies [12]. Though studies have compared T2N0 patients receiving neoadjuvant therapy and upfront surgery, they did not find any difference in outcomes except for the registry study from the Netherlands that favored neoadjuvant therapy [22, 36–40], but these studies have included both low- as well as high-risk early-stage tumors. A randomized controlled trial by Mariette et al. compared neoadjuvant chemoradiation followed by surgery versus surgery alone in stage I and II esophageal cancer patients. Though the trial was closed prematurely in view of being unlikely to show superiority in either arm, and the three-year overall survival was similar in both groups, postoperative mortality was significantly higher in the group receiving neoadjuvant treatment, i.e., 11.1% versus 3.4% [41]. Identifying factors predictive of high risk of recurrence and metastases will help to personalize the multimodality approach in patients with early esophageal cancer who are most likely to benefit.

Our study has its limitations associated with retrospective studies. EUS was performed only in tumors that were low volume on imaging and not in all patients who underwent surgery. The low number of patients with early-stage esophageal cancer in our study makes it difficult to draw definitive conclusions, but guides oncologists towards designing large-scale prospective trials with multi-institutional collaboration for this group of patients given the rising incidence of early esophageal carcinoma and wide-spread availability of endoscopic ultrasound. The strengths of this study include being conducted at a tertiary high-volume cancer center, discussion of all patients in a multidisciplinary tumor board at every step of the management pathway, and adherence to evidence-based guidelines (Table 5).

**Table 5:** Comparison with studies on EUS-staged early esophageal carcinoma

Variable	Current study	Nucci et al. [21] (cT2N0)	Atay et al. [22]	Haisley et al. [27]	Shin et al. [24]
***N***	68	72	499	110	240
Study period	2011–2019	2011–2018	2000–2015	2000–2016	2005–2010
Squamous	63	8	55	4	235
Adenocarcinoma	5	64	444	106	5
Upper 1/3	0	7	7	0	10
Middle 1/3	36	17	29	15	126
Lower 1/3 and GEJ	32	48	463	95	104
EUS accuracy					
T1	81.6%	NA	NA	79.1% (overall	96%
T2	46.7%	70.4%	24%	T stage)	22.7%
*N*	82.4%	79.1%	61%	91.8%	78%
Factors predicting understaging	T2 on EUS, longitudinal size > 3.5 cm, SUVmax > 3.05 on PETCT	GE junction, tumor length > 3 cm, grade 3	Tumor length > 3.25 cm, LVI	LVI, signet ring features. T2 stage	Poorly differentiated tumor, SUV max > 3.15
Survival	3 year	NA		5 year	NA
Not upstaged	81.3%		57% (5 year)	83.9%	
Understaged	48.1%		50% (2 year)	50.2%	

## Conclusion

Current staging modalities have limitations and can result in almost one-fifth of early esophageal cancers on imaging being upstaged on final histopathology. Specific high-risk factors like T2 stage, tumor length > 3.5 cm, and SUVmax > 3.05 can aid as a guide to identifying patients at risk of being locally advanced or having nodal metastases. Neoadjuvant therapy has been shown to improve outcomes in the locally advanced stages of esophageal cancer but it merits exploration in early-stage esophageal cancers with high risk of upstaging. Considering a multicentric prospective trial in this setting would help to identify patients most likely to benefit from multimodality approaches.

## Data Availability

No datasets were generated or analysed during the current study.
